# Marketing Strategies and Factors Influencing the Popularity of Alcohol Videos from Official Brand Accounts on Douyin: Content Analysis Study

**DOI:** 10.2196/74221

**Published:** 2026-01-06

**Authors:** Yuchen Zhao, Lingyun Zhang, Chenyu Qian, Wenjie Guo, Weiyun Zhu, Pinpin Zheng

**Affiliations:** 1 Department of Preventive Medicine and Health Education, School of Public Health, Institute of Health Communication, Fudan University, 138 Yixueyuan Road, Xuhui District, Shanghai, 200032, China, 86 13917249701; 2 Party Committee Office, Zhongshan Hospital Affiliated with Fudan University, Shanghai, China

**Keywords:** alcohol marketing, Douyin platform, alcogenic environments, advertisement, health warning

## Abstract

**Background:**

Alcohol consumption in China poses significant public health challenges. Alcohol marketing has been shown to increase public alcohol consumption, with social media platforms such as Douyin (TikTok in Mainland China) being among the main channels for alcohol marketing.

**Objective:**

This study aimed to analyze the thematic content of alcohol advertising on the Douyin platform and to explore the factors influencing the popularity of these types of advertising.

**Methods:**

Using data from the JINGDONG platform and alcohol industry reports, we identified 40 popular alcohol brands. For each brand, we located their official Douyin accounts and selected the top 20 most-liked videos posted between November 1, 2020, and November 1, 2021. In total, 659 videos from 37 brands were collected for analysis. Two trained researchers independently coded each video using a predefined codebook, which consisted of 7 sections and 20 items. Binary logistic regression was conducted with the grouping of the number of likes as the dependent variable, and the marketing strategies and warning elements of each video as independent variables.

**Results:**

Among the 659 videos analyzed, 320 (48.6%) garnered more than 1000 likes. A significant portion of the videos was direct advertisements (281/659, 42.6%) and short skits (255/659, 38.7%), with 56.0% (369/659) featuring characters engaging in drinking-related behaviors or directly consuming alcohol. Additionally, many videos highlighted brand elements (510/659, 77.4%) and extended features (161/659, 24.4%). Cultural themes were also common, with 23.2% (153/659) of the videos promoting the enjoyment of life and 6.8% (45/659) emphasizing balance in life. However, age restrictions were missing for 26.9% (177/659) of the videos, and only 1.2% (8/659) included a health warning stating that “Drinking is harmful to health.” Certain marketing strategies were significantly associated with greater video popularity, including the use of short skits (odds ratio [OR] 2.77, 95% CI 1.42‐5.41), highlighting brand elements (OR 2.96, 95% CI 1.59‐5.51), and emphasizing life balance (OR 3.44, 95% CI 1.11‐10.66). In contrast, the presence of age restrictions (OR 0.32, 95% CI 0.15‐0.67) and explicit health warnings (OR 0.06, 95% CI 0.01‐0.84) were associated with lower popularity. The period from July to September and November was the peak release period for alcohol advertisements on Douyin.

**Conclusions:**

Alcohol marketing strategies on Douyin leverage experiential, brand-driven, collaborative, and cultural marketing techniques to enhance video attractiveness and create alcogenic environments. Moreover, effective age restrictions and health warnings are largely absent. It is essential to legislate and enforce stricter alcohol marketing regulations to reduce the health risks associated with alcohol marketing.

## Introduction

Alcohol consumption is a leading risk factor for the global disease burden, which is associated with the risk of more than 200 diseases and injuries, causing approximately 3 million deaths globally each year, accounting for 5.3% of all deaths [1]. There is growing evidence that the public’s alcohol consumption could be influenced by “alcogenic environments,” which are settings in which alcohol is easily accepted, available, and affordable [2]. “Alcogenic environments” have been shown to be associated with harmful drinking behaviors, including alcohol addiction and underage drinking [3-5].

As an important part of the “commercial determinants of health,” alcohol marketing is a key driver for creating an alcogenic environment [2,5]. The alcohol industry often promotes products through intensive advertising and even emphasizes the “function” of drinking [3]. This marketing strategy of widespread advertising could help enhance the acceptability and normalization of alcohol consumption at both the individual and community levels, resulting in an increase in the alcogenic environment [5-8].

The widespread use of digital media has provided new channels for alcohol marketing, such as websites, apps, and social media [9,10]. Alcohol marketing on social media platforms has grown rapidly over the past two decades, with the alcohol industry reporting that alcohol marketing on social media platforms (including YouTube and Facebook) has reached many more consumers [11]. For instance, a previous study revealed that music videos on YouTube conveyed approximately 10.06 billion alcohol-related impressions to the British population [12].

As a country with high alcohol consumption, alcohol marketing on social media in China is also severe [13]. The annual advertising investment of the Chinese liquor industry has exceeded 20 billion yuan (approximately US $3 billion), with the goal of creating an all-media marketing platform combining television, websites, and social media [14]. A Chinese alcohol industry report in 2021 informed that 89% of alcohol consumers received alcohol-related information through the internet, with more than 30% coming from short video platforms such as Douyin (TikTok in mainland China) [15].

Douyin, China’s leading short video platform with more than 800 million daily active users, is primarily youth-centric, with 60% of its users aged younger than 30 years [16]. It has become a major hub for entertainment, social interaction, and marketing, leveraging algorithm-based recommendations to help users discover tailored content and enabling businesses to reach a broad audience with creative campaigns. Under the “Chinese liquor” tag, videos related to Chinese liquor on Douyin have a total of 65.63 billion views, with the most popular video receiving as many as 878,000 likes [17]. These videos feature diverse drinking scenes and emotions, using promotional strategies, such as collaborations with opinion leaders and brand partnerships [18].

According to the attention-interest-desire-action (AIDA) model, the consumer purchase decision-making process is characterized by a progression through four stages [19]: attention, interest, desire, and action. Previous research has demonstrated that higher levels of alcohol social media marketing exposure are associated with positive drinking expectancies and drinking behaviors [20,21], confirming that the AIDA model can be used to explain alcohol advertising exposure, where attention attracted by alcohol advertisements can translate into drinking behavior. However, regarding the “A (attention)” and “I (interest)” components of the AIDA model, likes can be viewed as a preliminary quantitative indicator of successful “interest” capture, and it remains unclear from previous research what types of alcohol-related social media advertisements effectively capture audience attractiveness.

The World Health Organization (WHO) has called for interventions to reduce the alcogenic environment, and the key interventions include restrictions on alcohol marketing [2]. However, China lacks comprehensive regulations governing social media alcohol advertising. It is essential to determine the marketing strategies of alcohol products to formulate more comprehensive regulations and supervision measures.

This study aimed to identify the marketing strategies of alcohol advertisements and the placement of warnings on the Douyin platform, the factors associated with the attractiveness of those alcohol advertisements, and the time trend of these alcohol videos. These findings may contribute to extending the application of the AIDA model in social media alcohol advertising, with a specific focus on exploring the mechanisms through which different types of alcohol marketing strategies facilitate the transition from the “attention” to the “interest” stage. These findings are also expected to provide policymakers with insights into how alcohol products are promoted and marketed on social media, which is crucial for enhancing the regulation of alcohol marketing.

## Methods

### Sampling and Data Collection

In 2021, the size of the online market for alcohol in China reached 136.31 billion yuan [22], of which JINGDONG [23] was one of the main Chinese e-commerce platforms. The sales rankings of alcohol brands on JINGDONG can reflect the popularity of those brands. By reviewing alcohol industry reports, we categorized the alcohol currently sold in China into six groups: Chinese liquor (spirits), beer, wine, yellow rice wine, fruit wine, and premixed cocktails. On the basis of this classification, we determined the alcohol brands for this study as follows:

- Database A: According to retail data from the JINGDONG platform in 2021, the top 5 alcohol brands in each category were selected, resulting in a total of 30 brands representing popular choices across all age groups. These brands represent products commonly purchased by general consumers.
- Database B: In addition to the brands aforementioned, some new brands are very popular among young people and women but lack large sales records. We identified the 10 most popular brands targeting these demographics by reviewing industry reports on alcohol consumption by young people and women.
- Combining databases A and B, a total of 40 brands were included in the study. We then identified the official Douyin accounts for each brand. Three brands did not have official Douyin accounts, leaving 37 brands for inclusion (Table 1). Given the similar formats of Douyin advertisements within the same brand and for the feasibility of the study, we selected the top 20 most-liked videos from each account that were released between November 1, 2020, and November 1, 2021. For brands that had released fewer than 20 videos in the past year, all available videos were included. The selection of 2021 as the data collection window was based on a key transition in Douyin’s commercialization process: before 2020, Douyin’s strategy focused primarily on user acquisition, while from 2020 onward, its commercialization accelerated, with the platform achieving the top position in domestic advertising revenue that same year [24]. This makes 2020‐2021 a suitable period for examining the marketing strategies of alcohol brands during the platform’s commercialization push. In total, 659 videos were collected for further analysis.

**Table 1.** Samples of alcohol brands in this study.

Categories	Brands
Chinese liquor	MOUTAI, WuLiangYe, Luzhou Laojiao, Yanghe, Fen Jiu, and Jiang Xiaobai
Beer	Budweiser, Tsingtao, Snowflake, Yanjing, Harbin, and Corona
Yellow or rice wine	Guyuelongshan, Kuaijishan, Jimo rice wine, Nverhong, MIK, Suzhou Qiao
Wine	Penfolds, Greatwall, Jacob’s Creek, Lafite, Chateau Monlot, Changyu/Torre Oria
Fruit wine or preconditioning of cocktails	Jin liquor, Jinro, UMEET, Huatian Xiangz, RIO, Breezer, Power Station, Jiushilang
Imported liquor	Absolut Vodka, Bacardi, Johnnie Walker, Ballantine, and Hennessy

### Coding Method

Codebook development was a top-down or bottom-up process. This process included the following phases. First is the top-down phase—reviewing the previous studies and the related advertising marketing reports [9,25,26], which allowed us to identify the marketing strategies used by official alcohol accounts on Douyin. Afterward, a preliminary framework of the codebook was developed with 8 main dimensions, including basic information, content presentation, scene setting, brand and product appeal, promotion strategy, emotion, culture, and warning (Multimedia Appendix 1). Second is the bottom-up phase—reviewing 20 randomly selected videos of official alcohol accounts on Douyin, which helped us identify the strategies that were undetermined in the preliminary framework. Accordingly, we enriched the codebook with subdimensions derived from the video content, including the specific form of the presentation, the characters’ drinking action, the scene, the product promotion strategy, the emotional tone, the cultural appeal, and different types of warnings. Third, 20 videos were randomly selected from the remaining samples for retested coding to verify the applicability of the current codebook. The resulting codebook consisted of 8 sections with 19 items, including basic information, content presentation, scene setting, brand and product appeal, promotion strategy, emotion, culture, and warning (Table 2).

Apart from the aforementioned elements, this research also collected the like counts of each video as the variable of video attractiveness. As 1000 is close to the median number of likes (Median=870, IQR: 135-7612), we categorized the number of likes into a high likes group and a low likes group using 1000 as the cutoff point.

The content analysis of the alcohol-related video started in December 2021 and took 1 month in total. A total of 659 videos were monitored. A total of 3 public health researchers participated in the coding work, and all researchers received training to unify the coding standards before the start of coding. After the training, the consistency of the researchers when coding the same video reached a kappa value of 92.8%, indicating a high level of consistency in their coding standards.

The specific coding work was conducted via an online questionnaire platform “Wenjuanxing.” The codebook was preentered into the platform and presented in the form of choice questions. Researchers selected the corresponding options on the online questionnaire platform while viewing Douyin videos. All videos were downloaded to local computers and coded independently by 2 researchers. If there were inconsistencies, the 2 researchers were required to rereview the video to confirm it. If they still failed to reach an agreement, the third researcher was involved in the discussion to reach a consensus.

**Table 2.** Coding book of alcohol videos on the Douyin platform.

Dimensions, variable, and category	Definition or example
Basic information	
Durations	
≤30	—^a^
31-60	—
≥61	—
Brand category	
Traditional	Brands from database A
New	Brands from database B
Content presentation	
Form of presentation	
Advertisement	A direct promotional message aimed at selling a product
Short skit	A brief video performance using theatrical elements
Vlog	Short-form documentaries
Film and TV^b^ show excerpts	The clips from film and TV shows
Other	Other forms
Characters’ drinking action	
No characters and no drinking behavior	No characters appear
Characters appear without drinking behavior	Characters do not touch alcohol products
Characters appear with drinking-related behavior	Characters hold alcohol glasses or pour alcohol and clink glasses
Characters appear with drinking behavior directly	Characters drink alcohol directly
Scene setting	
Drinking alone	Contain scenes of drinking alone
Party	Contain scenes of drinking at parties
Natural scenery	Contain scenes of natural scenery
Cultural or sports activities	Contain scenes of cultural or sports activities
Brand and product appeal	
Brand elements	Contain brand name, brand logo, or brand mascot
Product elements	Contain product name or product logo
Intrinsic product features	Contain information about the odor, color, taste, and materials of the product
Extended product features	Contain information about the origin, production process, vintage, and creative drinking methods
Promotion strategy	
Product promotion strategy	
Key opinion leaders	Invite celebrities to endorse
Cross-border brand cooperation	Collaborate with other brands to promote
Interaction with audience	Engage with the audience to attract fans
Not mentioned	—
Cues refer to women’s interests	Contain cues that refer to women’s interests, such as flowers, perfume
Cues refer to youth’s interests	Contain cues that refer to youth interests, such as cartoons, cosplay
Emotion	
Emotional tone	
Positive	A favorable or optimistic emotion conveyed in the video, such as pleasure, moving
Neutral	An emotion neither leans positive nor negative, such as calmness
Negative	An unfavorable or unpleasant emotion conveyed in the video, such as sadness, loss
Culture	
Cultural appeal	
Historical inheritance	Highlighting the alcohol’s history, heritage, and tradition
Festival celebration	Emphasizing drinking as a way to celebrate the holiday
Balance in life	Emphasize the philosophical concept of balance in life gained through drinking
Ambition and striving	Drinking symbolizes ambition and striving for success
Enjoy one’s life	Emphasizing drinking as a way to enjoy life
Not mentioned	—
Warning	
Age restriction	
Yes or no	Contain age restrictions, such as “minors under the age of 18 are prohibited from drinking alcohol”
Health warnings	
Drinking is harmful to health	Contain health warning related to “drinking is harmful to health”
Please drink responsibly	Contain health warning related to "please drink responsibly”
Not mentioned	—

a Not applicable.

b TV: television.

### Statistical Analysis

Frequencies and proportions are reported for the basic information, the marketing elements, and the warnings of Douyin videos. Chi-square tests were conducted to examine the association between different marketing strategies and the grouping of like counts, as well as the association between the warnings in videos and the grouping of like counts. As the dependent variable, “grouping of like counts,” is a binary variable, we conducted a binary logistic regression, with the low-likes group serving as the reference. The marketing elements and warning elements of each video, which were recorded through coding, served as the independent variables. The enter method was adopted for the model to explore the factors affecting the popularity of Douyin videos among the public.

Adjusted odds ratios (ORs) and their 95% CIs were used to quantify the effects. To evaluate the model’s goodness of fit, the Nagelkerke R² was used to determine the explanatory power of the model. To investigate the seasonal variations in Douyin videos, this study compiled the release times of the sampled videos and generated a scatter plot depicting the monthly distribution of video releases. IBM SPSS software (version 20.0) was used to carry out all the analyses.

### Ethical Considerations

According to Article 32 of China’s National Health Commission, Ministry of Education, and Ministry of Science and Technology Document No. 4 in 2023 “Notice on Issuing the Measures for Ethical Review of Human Life Science and Medical Research,” research using legally obtained publicly available data, data generated through observation without interfering with public behavior, or anonymized information data is exempt from ethical review [27]. This study uses legally accessible public data from social media platforms, involves no individual user data, and does not interfere with public behavior. Therefore, this study qualifies for an exemption from ethical review.

## Results

### The Marketing Strategies of Alcohol Advertisements and the Placement of Warnings

Among the 659 Douyin videos analyzed, the number of likes ranged from 2 to 440,000, with a median of 870 (IQR: 135-7612). Using 1000 likes as the cutoff point, 320 videos (48.6%) were classified as high-liked videos, with more than 1000 likes, whereas 339 videos (51.4%) were classified as low-liked videos, with fewer than 1000 likes.

**Figure 1.**
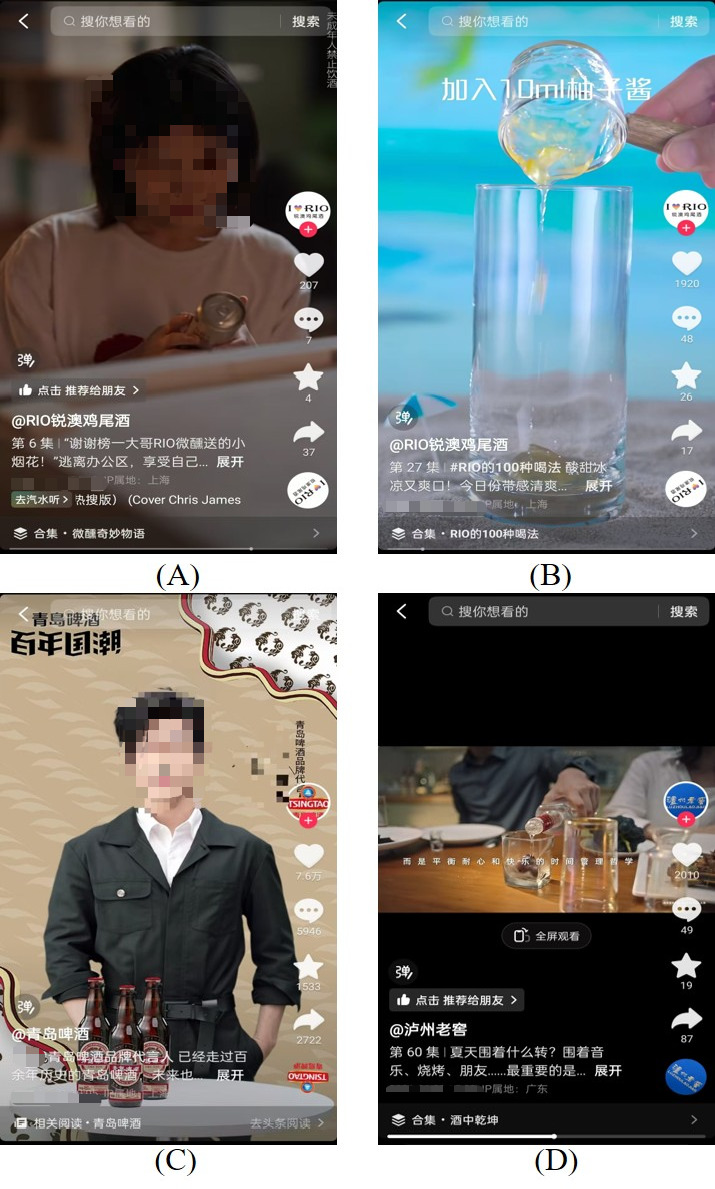
Screens depicting the marketing strategy for alcohol advertisements on the Douyin platform. (A) Use of a short skit. (B) Creative method of drinking the product (adding grapefruit sauce to a cocktail). (C) Use of key opinion leaders. (D) The promotion of a life balance philosophy (drinking is a time management philosophy that balances patience and happiness).

Most videos were presented as advertisements (n=281, 42.6%) and short skits (n=255, 38.7%), with 56.0% (n=369) of the characters engaging in drinking-related behavior or drinking directly. The most frequent scenes were parties (n=170, 25.8%) and natural scenery (n=112, 17.0%). A total of 254 (38.5%) videos showed the product’s intrinsic features, such as taste and odor, whereas 161 (24.4%) videos highlighted extended features, such as creative ways of mixing and drinking Rio cocktails with Sprite. Some videos conveyed drinking-related culture, mainly including enjoying one’s life (n=153, 23.2%) and historical inheritance (n=65, 9.9%). For example, “Rainy days go better with blueberry wine” and “Tradition is our unwavering craftsmanship” (Figure 1).

Additionally, 36.6% (n=241) of the videos included elements favored by women, such as flowers and perfume, and 16.1% (n=106) contained elements appealing to teenagers, including e-sports and anime. However, not all videos included age restrictions (n=482, 73.1%), and only 1.2% (8/659) contained health warnings “Drinking is harmful to health” (Table 3).

**Table 3.** Characteristics comparison of alcohol videos with different popularities.

Dimensions, variable, and category	Values, n (%)	Low likes, n (%)	High likes, n (%)	Chi-square (*df*)	*P* value
Basic Information					
Durations				12.1 (2)	.002
0-30	340 (51.6)	184 (54.3)	156 (48.8)		
31-60	150 (22.8)	87 (25.7)	63 (19.7)		
>60	169 (25.6)	68 (20.1)	101 (31.6)		
Brand category				9.6 (1)	.002
New	299 (45.4)	134 (39.5)	165 (51.6)		
Traditional	360 (54.6)	205 (60.5)	155 (48.4)		
Alcohol category				161.6 (5)	<.001
Chinese liquor	118 (17.9)	42 (12.4)	76 (23.8)		
Beer	111 (16.8)	8 (2.4)	103 (32.2)		
Wine	103 (15.6)	80 (23.6)	23 (7.2)		
Imported liquor	74 (11.2)	53 (15.6)	21 (6.6)		
Fruit wine or preconditioning of cocktails	134 (20.3)	69 (20.4)	65 (20.3)		
Yellow or rice wine	119 (18.1)	87 (25.7)	32 (10.0)		
Content presentation					
Form of presentation				7.6 (4)	.11
Advertisement	281 (42.6)	147 (43.4)	134 (41.9)		
Short skit	255 (38.7)	122 (36.0)	133 (41.6)		
Film and television show excerpts	20 (3.0)	7 (2.1)	13 (4.1)		
Vlog	70 (10.6)	42 (12.4)	28 (8.8)		
Other	33 (5.0)	21 (6.2)	12 (3.8)		
Characters’ drinking action				28.1 (3)	<.001
No characters and no drinking behavior	106 (16.1)	79 (23.3)	27 (8.4)		
Characters appear without drinking behaviors	184 (27.9)	92 (27.1)	92 (28.8)		
Characters appear with drinking-related behaviors	265 (40.2)	119 (35.1)	146 (45.6)		
Characters appear with drinking behaviors	104 (15.8)	49 (14.5)	55 (17.2)		
Scene setting					
Drinking alone				3.1 (1)	.08
Yes	99 (15.0)	59 (17.4)	40 (12.5)		
No	560 (85.0)	280 (82.6)	280 (87.5)		
Party				16.0 (1)	<.001
Yes	170 (25.8)	65 (19.2)	105 (32.8)		
No	489 (74.2)	274 (80.8)	215 (67.2)		
Natural scenery				0.6 (1)	.45
Yes	112 (17.0)	54 (15.9)	58 (18.1)		
No	547 (83.0)	285 (84.1)	262 (81.9)		
Cultural or sports activities				3.5 (1)	.06
Yes	75 (11.4)	31 (9.1)	44 (13.8)		
No	584 (88.6)	308 (90.9)	276 (86.3)		
Brand and product appeal					
Brand elements				45.9 (1)	<.001
Yes	510 (77.4)	226 (66.7)	284 (88.8)		
No	149 (22.6)	113 (33.3)	36 (11.3)		
Product elements				2.0 (1)	.16
Yes	399 (60.5)	214 (63.1)	185 (57.8)		
No	260 (39.5)	125 (36.9)	135 (42.2)		
Intrinsic product features				48.2 (1)	<.001
Yes	254 (38.5)	174 (51.3)	80 (25.0)		
No	405 (61.5)	165 (48.7)	240 (75.0)		
Extended product features				28.0 (1)	<.001
Yes	161 (24.4)	112 (33.0)	49 (15.3)		
No	498 (75.6)	227 (67.0)	271 (84.7)		
Promotion strategy					
Product promotion				22.7 (3)	<.001
Key opinion leaders	53 (8.0)	14 (4.1)	39 (12.2)		
Cross-border brand cooperation	32 (4.9)	23 (6.8)	9 (2.8)		
Interaction with audience	12 (1.8)	3 (0.9)	9 (2.8)		
Not mentioned	562 (85.3)	299 (88.2)	263 (82.2)		
Cues refer to women’s interests				4.4 (1)	.04
Yes	241 (36.6)	111 (32.7)	130 (40.6)		
No	418 (63.4)	228 (67.3)	190 (59.4)		
Cues refer to teenagers’ interests				20.5 (1)	<.001
Yes	172 (26.1)	63 (18.6)	109 (34.1)		
No	487 (73.9)	276 (81.4)	211 (65.9)		
Emotion					
Emotional tone				3.0 (2)	.22
Positive	263 (39.9)	128 (37.8)	135 (42.2)		
Neutral	359 (54.5)	195 (57.5)	164 (51.3)		
Negative	37 (5.6)	16 (4.7)	21 (6.6)		
Culture					
Cultural appeal				40.4 (6)	<.001
Historical inheritance	65 (9.9)	43 (12.7)	22 (48.8)		
Festival celebration	49 (7.4)	35 (10.3)	14 (6.9)		
Balance in life	45 (6.8)	8 (2.4)	37 (4.4)		
Ambition and striving	33 (5.0)	17 (5.0)	16 (19.7)		
Enjoy one’s life	153 (23.2)	90 (26.5)	63 (11.6)		
Gift-giving culture	21 (3.2)	9 (2.7)	12 (5.0)		
Not mentioned	292 (44.5)	137 (40.4)	156 (3.8)		
Warning					
Age restriction				19.4 (1)	<.001
Yes	482 (73.1)	273 (80.5)	209 (65.3)		
No	177 (26.9)	66 (19.5)	111 (34.7)		
Health warnings				17.0 (1)	<.001
Drinking is harmful to health	8 (1.2)	6 (1.8)	2 (0.6)		
Please drink responsibly	68 (10.3)	50 (14.7)	18 (5.6)		
Not mentioned	583 (88.5)	283 (83.5)	300 (93.8)		

### The Factors Associated With the Attractiveness of Alcohol Advertisements

Compared with videos from traditional brands, videos from new brands were more likely to receive more likes (OR 1.759, 95% CI 1.041‐2.972). Compared with Chinese liquor videos, beer videos garnered more likes (OR 11.310, 95% CI 3.130‐40.873), whereas wine, imported liquor, and yellow or rice wine videos received fewer likes (OR 0.171, 95% CI 0.076‐0.385; OR 0.172, 95% CI 0.039‐0.753; and OR 0.077, 95% CI 0.030‐0.195).

**Figure 2.**
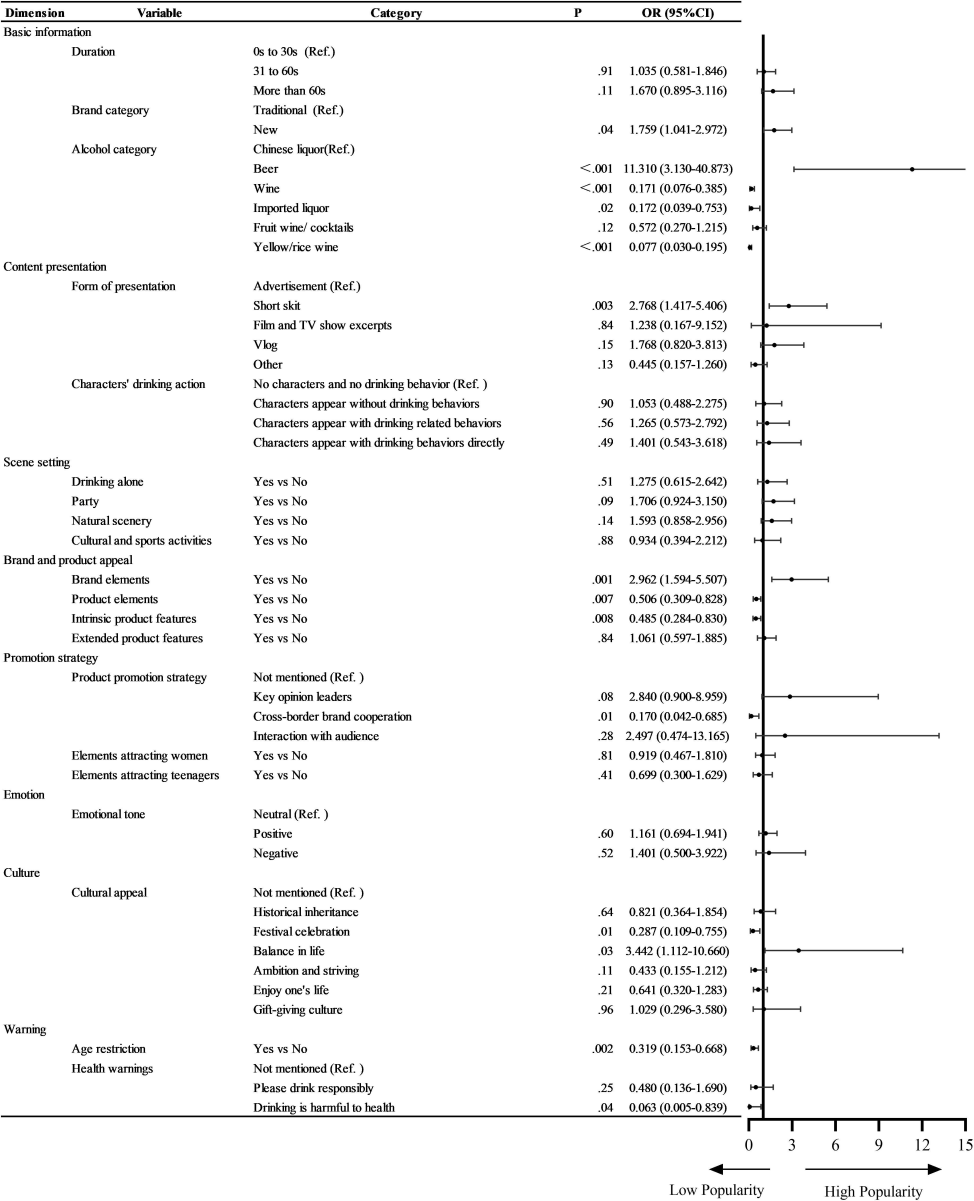
Multivariate logistic regression of popularity of alcohol-related videos. OR: odds ratio.

Compared with direct advertisements, videos presented as short skits were more likely to receive likes (OR 2.768, 95% CI 1.417‐5.406). Videos conveying the culture of “balance in life” received more likes (OR 3.442, 95% CI 1.112‐10.660). Additionally, videos that displayed brand-related information were more attractive (OR 2.962, 95% CI: 1.594‐5.507), whereas those showcasing product elements or intrinsic features garnered fewer likes (OR 0.506, 95% CI 0.309‐0.828; and OR 0.485, 95% CI 0.284‐0.830). Furthermore, videos with age restrictions and the health warning “Drinking is harmful to health” tended to receive fewer likes (OR 0.319, 95% CI 0.153‐0.668; OR 0.063, 95% CI 0.005‐0.839). The total Nagelkerke *R*² of this logistic regression was 0.561 (Figure 2).

### Temporal Trend of Alcohol Video Releases on Douyin

**Figure 3.**
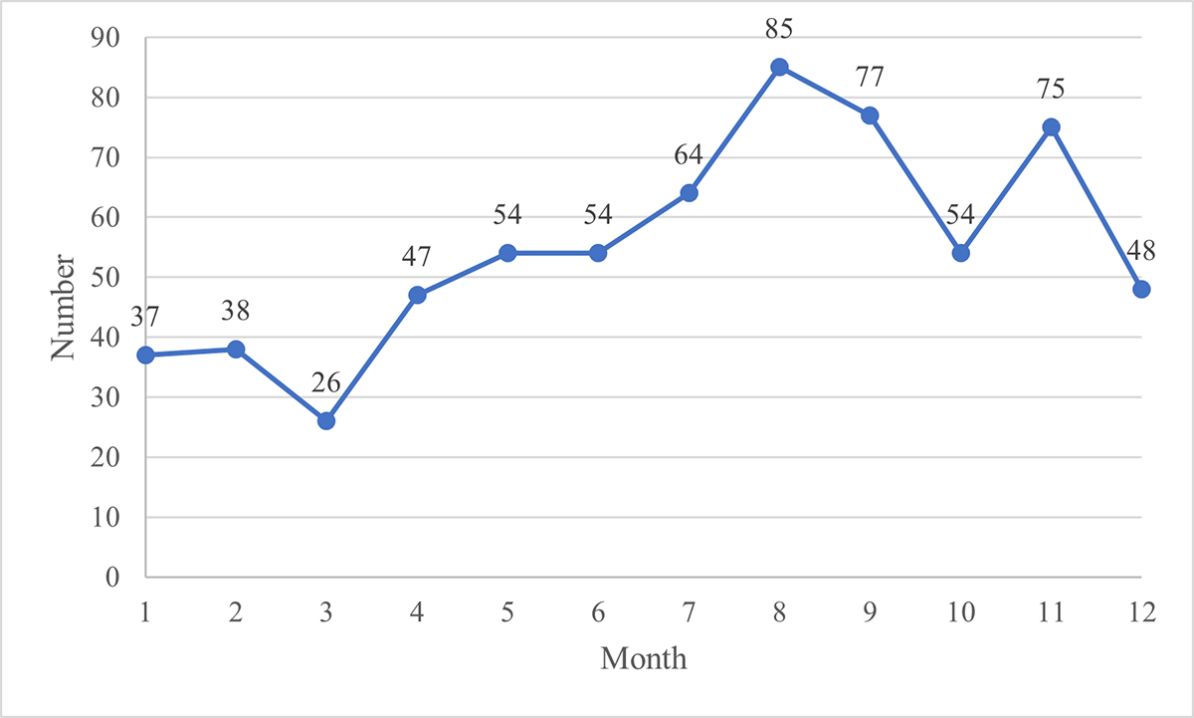
Distribution of Douyin alcohol advertisement release times.

The period from July to September of that year was the peak season for alcohol advertisements on Douyin, with the highest monthly number reaching 85. In addition, a release peak for Douyin alcohol videos was recorded in November, with the number reaching 75 (Figure 3).

## Discussion

### Principal Findings

To the best of our knowledge, this is the first study to analyze the thematic content of alcohol advertising on the Douyin platform. Through the analysis of 659 Douyin videos, we identified several alcohol marketing strategies, including diverse forms of presentation, highlighting the brand elements and incorporating cultural elements into the videos. In addition, we explored the factors influencing the popularity of those videos and reported that the use of several marketing approaches was positively correlated with the attractiveness of the videos. All these alcohol marketing practices may accelerate public alcohol consumption through enhancing alcogenic environment. These findings emphasize the urgent need for strong policy formulation and enforcement to reduce the negative influence of alcohol marketing on social media platforms.

### Alcohol Types and Temporal Trends

According to the results, beer advertisements were more popular than Chinese liquor advertisements, whereas wine, imported liquor, and rice or yellow wine advertisements received less attention. We speculate that this is due to the high overlap between the characteristics of the beer-consuming audience and Douyin users. According to the Douyin user portrait report, the main audience of Douyin consists of young people aged 19 to 30 years from non–first-tier cities [28]. Chinese youth primarily prefer beer, followed by Chinese liquor, whereas wine, imported liquor, and yellow wine are far less popular [29]. The temporal trend of Douyin alcohol videos also reflects young Chinese people’s preferences. Unlike the traditional peak season of Chinese liquor during the Spring Festival season (December to February) [30], alcohol advertisements on Douyin are heavily launched during the summer vacation period (July to September) as well as the month of the Double 11 Shopping Festival to cater to young people’s demand for beer consumption. As in previous studies, all these factors indicate that social media platforms such as Douyin have become popular alcohol marketing channels targeting youth [31-33].

### Alcohol Marketing Strategies on Douyin

The first alcohol marketing strategy on the Douyin platform is experiential marketing, a prime example of which is the utilization of short skits [34]. Unlike traditional advertising, these skits occur within an entertainment context, becoming part of an experience that immerses the viewer in the storyline [35]. This approach may blur the lines between audiences’ personal lives and commercial messages while frequently depicting alcohol-related behaviors, which may encourage higher drinking frequency among viewers [31,36,37]. Another marketing strategy is brand marketing, which emphasizes brand elements such as the logo or mascot, rather than simple introductions of products. These elements could convey brand emotions and values, prompting people to hold a positive attitude toward a brand or product, thus increasing the probability of purchasing [38,39]. Brand marketing is not only common in China but has also been validated by previous studies as one of the prevalent strategies in global alcohol marketing [40].

The third strategy is collaborative marketing, in which the involvement of celebrities becomes the main strategy. Positive characteristics associated with the celebrity can be transferred to the product. In particular, the celebrity endorsement of young people could increase their recollection of drinking images [32,41]. In addition, many alcohol brands choose to collaborate with other brands or activities, including the National Basketball Association and music festivals, which could also broaden their audience beyond traditional alcohol consumers. The last strategy is cultural marketing. Many alcohol brands incorporate cultural elements into their promotions [42]. Similar to previous studies, this is also the most commonly used marketing strategy in alcohol advertisements on Chinese television [43]. Previous studies have demonstrated that adapted cultural value appeals are more persuasive and attractive in advertisements [44]. On the other hand, alcohol advertising could further strengthen the “alcohol culture,” enhance the alcogenic environment, and ultimately promote alcohol consumption.

This study extends the application of the AIDA model to alcohol advertising on the Douyin platform. Multivariate analysis revealed that when audiences are exposed to alcohol videos using experiential, brand, collaborative, and cultural marketing strategies, these videos are more likely to gain audience likes, thereby facilitating the transition from “attention” to “interest.” To prevent the transition of audience attention to purchasing action, reducing the use of these marketing strategies in alcohol videos is crucial, thereby limiting the formation of “interest.”

### Age Restriction and Health Warning

Notably, many videos include cues related to teenagers’ interests, such as idols, cartoons, and e-sports. Although there is currently no regulation explicitly requiring alcohol advertisements to include age restriction warnings, Douyin’s platform policy clearly mandates that alcohol advertisements must contain warnings such as “Users under the age of 18 are prohibited from purchasing this product.” [45] However, not all videos contain those age restriction signs. Although the presence of age restrictions may reduce the number of likes, the actual effectiveness of these restrictions remains unclear. It is generally easy to access social media platforms, and even children younger than 13 years can “legally” use social media and view alcohol advertisements, despite these age restrictions in place [46]. It is urgent to standardize the “teenage mode” on these social media platforms to mitigate the influence of alcohol marketing on teenagers.

Moreover, the lack of health warnings on social media platforms is a serious issue. Only 11.5% of the videos include any form of health warning, and most of these are ambiguous messages, “drink responsibly.” In fact, “drink responsibly” messages are associated with increased alcohol consumption [47]. This is because such messages tend to enhance a prodrinking social norm. When “responsible drinking” messages are placed in alcohol advertisements, the alcohol industry can give the impression of fulfilling corporate responsibility without decreasing sales [48,49]. As reported in previous studies, only explicit health warnings that inform consumers about the carcinogenic effects of alcohol have a significant effect, rather than ambiguous messages [49]. Consequently, the 2022‐2030 WHO Global Alcohol Action Plan has called for ensuring that the labeling of alcoholic beverages is appropriate [50] and that essential health protection information is displayed.

### Policy Implications

Although China’s Advertising Law stipulates that all alcohol advertisements must not encourage drinking, depict drinking behaviors, or emphasize the functional benefits of alcohol consumption, and additionally prohibits the dissemination of alcohol advertisements through mass media targeting minors [51], alcohol marketing remains highly prevalent on social media platforms such as Douyin, where minors can still be easily exposed to such content. This suggests that deficiencies in both the legal provisions on alcohol advertising and their enforcement remain.

With respect to laws governing alcohol advertising, first, the definition of alcohol advertising should be refined to cover not only direct advertisements but also embedded stealth advertising, such as short skits and placements. Thus, legislative restrictions should include a comprehensive ban on all forms of alcohol commercial communication, recommendation, or activity. While these factors may not directly encourage drinking, they could help enhance the alcogenic environment. Moreover, the definition of “media targeting minors” remains ambiguous, making it possible for minors to be exposed to alcohol advertisements through general-audience channels, especially social media [43]. It is imperative to either explicitly prohibit alcohol advertising on social media or establish a robust “teenage mode.” With respect to age restriction warnings and health warnings for alcohol advertising, mandatory regulations should not be limited to platforms such as Douyin but must be incorporated into advertising laws and strictly enforced. Finally, health warnings should be provided to avoid vague expressions such as “drink responsibly” and instead provide specific examples of the health risks associated with alcohol consumption.

With respect to law enforcement, the responsibility for monitoring and enforcing the existing regulations on advertising in China lies primarily with only the current commercial administrative department. In the future, other administrative departments, including health, food, and drug departments, could also participate to establish a comprehensive enforcement network [21]. Previous studies have demonstrated that the low penalties in the case of violation and the lack of effective detection are also key factors hindering its effective enforcement [21]. Enhancing penalty severity, improving regulatory channels, and encouraging public participation in supervision can significantly strengthen the enforcement of the law. With comprehensive legislation and implementation of restrictions, reducing the alcogenic environment caused by alcohol marketing is among the most cost-effective ways.

### Limitations

The data collection period for this study (2021) may present certain limitations. With the rapid evolution of the digital marketing environment, the promotional strategies of alcohol brands on social media platforms, such as Douyin, may have become more covert and innovative. In recent years, emerging approaches such as algorithm-driven personalized content placement, interactions with virtual spokespersons, and cross-platform integrated marketing campaigns have increased in prevalence. These strategies are often integrated more deeply into users’ everyday browsing experiences, further blurring the boundary between commercial promotion and organic content. Future monitoring of alcohol advertising should focus on such covert marketing techniques to mitigate the potential impact of alcogenic environments.

There are also several limitations of this study. First, this study only gathered alcohol advertisements from Douyin over a 1-year period, failing to gather data over an extended period to assess the changing trends in marketing strategies over the years. Future studies could conduct longer-term longitudinal research to analyze temporal trends of marketing strategies in alcohol advertising. Second, like counts were used as the only variable representing popularity. The specific expressions and interactions of the audience in the comment section were not included and need further exploration. Despite these limitations, this study lays a foundation for future research on alcohol marketing content on Chinese social media platforms and provides evidence for strengthening the regulation of alcohol marketing on social media platforms.

### Conclusions

In conclusion, this study summarized several alcohol marketing strategies on the Douyin platform, including experiential marketing, brand building, cross-border collaboration, and cultural connection. These strategies may enhance video attractiveness and appeal to teenagers, serving as key factors in transforming “attention” into “interest” (2 basic elements in the AIDA model) of alcohol advertisements. However, effective age restrictions and explicit health warnings are rarely shown in these alcohol-related Douyin videos. Urgent actions should include closing existing legal loopholes, such as refining the definition of alcohol advertising, strengthening protections for minors, and requiring specific health warnings, along with enhancing multiagency collaboration and imposing stricter penalties to decrease the alcogenic environment.

## Supplementary material

10.2196/74221 Multimedia Appendix 1 The primary coding book of alcohol videos on Douyin platform.

## Abbreviations

AIDA: attention-interest-desire-action
OR: odds ratio
WHO: World Health Organization
