# Supplementary material for: Marketing Strategies and Factors Influencing the Popularity of Alcohol Videos from Official Brand Accounts on Douyin: Content Analysis Study
Source: JMIR Infodemiology. 2026 Jan 6;6:e74221. doi: 10.2196/74221 (PMC12774395; doi:10.2196/74221)
Supplement: Multimedia Appendix 1 [file infodemiology-v6-e74221-s001.docx]

| **Table S1. The primary coding book of alcohol videos on Douyin platform** | | | |
| --- | --- | --- | --- |
| **Dimensions** | **Variable** | **Category** | **Defination/Example** |
| Basic information | |  |  |
|  | Duration (s) | ≤30; 31-60; ≥61 |  |
|  | Brand category | Traditional | Brands from database A |
|  |  | New | Brands from database B |
| Content Presentation |  |  |  |
|  | Form of presentation | **Undetermined** | The format or style used to present the video content |
|  | Characters' drinking action | **Undetermined** | Characters' interaction with alcoholic products |
| Scene setting | **Undetermined** |  | The background setting depicted in the video. |
|  |  |  |  |
| Brand and product Appeal |  |  |  |
|  | Brand elements | Yes or No | Contain brand name, brand logo or brand mascot |
|  | Product elements | Yes or No | Contain product name or product logo |
|  | Intrinsic product features | Yes or No | Contain information about the odor, color, taste and materials of product |
|  | Extended product features | Yes or No | Contain information about the origin, production process, vintage |
| Promotion strategy |  |  |  |
|  | Product promotion strategy | **Undetermined** | The specific marketing method used to promote the product |
|  | Cues refer to women’s interests | Yes or No | Contain cues that refer to women’s interests, such as flowers, perfume |
|  | Cues refer to youth’s interests | Yes or No | Contain cues that refer to youth’s interests, such as cartoon, cosplay |
|  |  |  |  |
| Emotion | Emotion tone | Positive | A favorable or optimistic emotion |
|  |  | Neutral | An emotion neither leans positive nor negative |
|  |  | Negative | An unfavorable or unpleasant emotion |
|  |  |  |  |
| Culture | Culture Appeal | **Undetermined** | Cultural elements to enhance cultural relevance or resonance. |
|  |  |  |  |
| Warning | Age restriction | Yes or No | Contain age restriction about alcohol |
|  | Health warnings | Yes or No | Contain health warning about alcohol |
